# Effect of Aortic Cross-Clamping Time on Development of Postoperative Atrial Fibrillation in Isolated CABG: A Single-Center Prospective Clinical Study

**DOI:** 10.21470/1678-9741-2022-0458

**Published:** 2023-06-14

**Authors:** Hatice Işil Dayi, Eyup Serhat Çalik, Oguzhan Birdal, Muhammed Enes Aydin, Ferhat Borulu, Ziya Yildiz, Bilgehan Erkut, Yahya Unlu

**Affiliations:** 1 Department of Cardiovascular Surgery, Erzurum City Hospital, Erzurum, Turkey; 2 Department of Cardiovascular Surgery, Faculty of Medicine, Ataturk University, Erzurum, Turkey; 3 Department of Cardiology, Faculty of Medicine, Ataturk University, Erzurum, Turkey; 4 Department of Anesthesiology, Faculty of Medicine, Ataturk University, Erzurum, Turkey

**Keywords:** Coronary Artery Bypass, Cardiopulmonary Bypass, Constriction, Atrial Fibrillation, Incidence

## Abstract

**Introduction:**

Many etiological factors affect the occurrence of atrial fibrillation after coronary artery bypass grafting. In this study, the relationship between cardiopulmonary bypass and cross-clamping times and the development of postoperative atrial fibrillation was examined.

**Methods:**

All patients who underwent isolated coronary artery bypass grafting with the same surgical team in our clinic between September 2018 and December 2019 were prospectively included in the study, and their perioperative data were recorded.

**Results:**

One hundred and three patients who met the specified criteria were included in the study. The median age was 62 (interquartile range: 54-71) years, and 82 (79.6%) were male. The patients were divided into two groups: those who developed atrial fibrillation and those who did not. Atrial fibrillation developed in 25 of 103 patients (24.3%). All patients underwent isolated coronary artery bypass grafting under standard cardiopulmonary bypass. The median duration of cardiopulmonary bypass was 72 (interquartile range: 63-97) minutes in those with atrial fibrillation and 82 (61-98) minutes in those without it, and there was no statistical difference (P=0.717). The median cross-clamping time was 40 (32.5-48) minutes in those with atrial fibrillation and 39.5 (30-46) minutes in those without it. Statistically, the relationship between cross-clamping time and atrial fibrillation was not significant (P=0.625).

**Conclusion:**

Our study found no significant relationship between cardiopulmonary bypass and cross-clamping times and the incidence of postoperative atrial fibrillation. However, we believe that there is a need for large-scale and multicenter clinical studies on the subject.

**Table:** 

Abbreviations, Acronyms & Symbols				
ACT	= Activated clotting time		IABP	= Intra-aortic balloon pump
AF	= Atrial fibrillation		ICU	= Intensive care unit
AFG	= Atrial fibrillation group		IQR	= Interquartile range
BMI	= Body mass index		K	= Potassium
Ca	= Calcium		LA	= Left atrial
CABG	= Coronary artery bypass grafting		LIMA	= Left internal mammary artery
CC	= Cross-clamping		Na	= Sodium
CPB	= Cardiopulmonary bypass		NAFG	= Non-atrial fibrillation group
DM	= Diabetes mellitus		PO₂	= Partial pressure of oxygen
ECG	= Electrocardiography		POAF	= Postoperative atrial fibrillation
EF	= Ejection fraction		RCA	= Right coronary artery
Hct	= Hematocrit		SD	= Standard deviation
Hgb	= Hemoglobin			

## INTRODUCTION

Atrial fibrillation (AF) after isolated coronary artery bypass grafting (CABG) is a complication accompanied by many clinical conditions with high morbidity and mortality, such as stroke, pulmonary embolism, extremity ischemia, and increase in hospital stay and treatment costs. Postoperative AF (POAF) is the most common complication after cardiac surgical procedures, occurring in 25% of patients after isolated CABG, 30% after isolated valvular procedures, 40-50% after combined valve replacement and CABG, and most frequently between the second and fourth days^[1,2]^. POAF maintains its status as an important clinical problem since its pathophysiology has not been fully elucidated. Therefore, an effective reduction in morbidity and mortality has not been achieved^[1]^.

The strongest risk factors associated with development of AF after CABG are advanced age, chronic obstructive pulmonary disease, respiratory complications, left atrial (LA) enlargement, right coronary artery (RCA) disease, male sex, previous cardiac surgery, preoperative atrial tachyarrhythmias, and elevated postoperative adrenergic tone^[2,3]^.

AF occurrences after cardiac surgery are usually seen in the first five days. In patients without a prior history of atrial arrhythmias, POAF usually revert to a sinus rhythm within six weeks of implementing an appropriate treatment strategy^[2]^. Despite multiple advances in surgery and perioperative care during the past two decades, no reduction in the incidence of POAF or associated complications has been achieved in clinical practice. Reducing the development of POAF by identifying other perioperative-related causes will enhance clinical recovery after surgery. In this study, we investigated the relationship between cardiopulmonary bypass (CPB) and cross-clamping (CC) times and development of POAF.

## METHODS

### Ethical Statement

The study protocol was accepted by the ethics committee of Atatürk University’s Faculty of Medicine (B.30.2.ATA.0.01.00), and the principles of the Declaration of Helsinki were followed (2013). All patients gave their written informed consent.

### Study Design, Population, and Criteria

This study was designed prospectively at the Department of Cardiovascular Surgery of Atatürk University’s Faculty of Medicine. This research recorded and analyzed 103 consecutive patients who underwent isolated CABG by the same surgical team between September 2018 and December 2019. Cases were included in the study if they underwent isolated CABG and used β-blockers before the operation, had normal thyroid function tests, and did not have valve disease. Patients were excluded from the study if they had preoperative AF or arrhythmia, underwent emergency operations (surgery on the day of the referral or the next day), had hypothyroid or hyperthyroidism, valve disease, or left ventricular aneurysm repair, underwent off-pump CABG, there was chronic steroid and non-steroidal anti-inflammatory drug use or preoperative use of digoxin, and had renal failure (serum creatinine > 2 mg/dl). Patients who did not use β-blockers in the preoperative period were included in the study by allowing them to start the drug at least 24 hours before the operation^[2]^.

### Predictors of Atrial Fibrillation

Potential predictors of AF were selected based on previous studies^[3-5]^. Preoperative variables were considered, including age, sex, LA diameter, ejection fraction (EF), diabetes, hypertension, smoking, body mass index (BMI), β-blocker use, and the laboratory parameters hemoglobin (g/dL), hematocrit (Hct) (%), and partial pressure of oxygen (PO₂) (mmHg). Intraoperative variables also were considered, including CC and CPB durations, myocardial and CPB temperatures, cardioplegia solutions (crystalloid, blood), use of the left internal mammary artery (LIMA), presence of RCA anastomosis, and number of distal anastomoses. Postoperative period AF predictors serum electrolytes and other laboratory data were determined as well as the duration of intubated follow-up and length of stay in the intensive care unit (ICU)^[3-5]^. Patients receiving antihypertensive treatment were defined as hypertensive. Two-dimensional transthoracic echocardiography imaging was performed with the appropriate windows and techniques before and after CABG. LA diameters and EF measurements were recorded.

### Protocols for Anesthesia and Surgery

The patients were prepared for CABG by applying standard anesthesia protocols. All patients were anesthetized with inhaler and intravenous narcotic anesthesia techniques. After a median sternotomy, LIMA and other grafts were prepared simultaneously. All patients were operated on under standard CPB. For heparinization, 3-5 mg/kg heparin was given according to the initial activated clotting time (ACT). An antegrade cardioplegia cannula was placed in the aortic root after aortic arterial and two-stage venous cannulation when the ACT value was between 400 and 650 seconds. Myocardial protection was provided by applying antegrade, hyperkalemic blood cardioplegia, and systemic hypothermia (32-34 °C) after aortic CC. Distal anastomoses were performed under CC. Proximal anastomoses were conducted under a side clamp placed on the aorta by removing the CC after all distal anastomoses were complete. When appropriate temperature and hemodynamics were achieved, decannulation was executed by weaning from CPB. After bleeding controls were provided, all patients whose surgical and anesthesia procedures were terminated by closing the sternum and skin incisions were taken to the ICU with the transport monitor.

### Follow-up after Surgery: Definition and Diagnosis of Atrial Fibrillation

Patients were extubated when their alertness and muscle strength returned to normal on the day of operation, and those who were hemodynamically stable were followed up in the ICU for two days. Continuous electrocardiography (ECG) follow-up was performed with standard D-II leads with a five-lead monitor in the ICU and on the first day of admission to the clinic. After ICU follow-up, clinically uneventful patients were taken to the clinic, and after the first 24 hours, their heart rate and arterial blood pressure were followed up at a maximum of four-hour intervals. Data of all patients were recorded postoperatively from the zero to the seventh day and documented by taking standard 12-lead ECGs every day.

After isolated CABG, any AF episode that lasted > 15 minutes at ECG monitoring or needed therapy for hemodynamic instability during hospitalization was defined as POAF^[6]^. AF was defined with 12-lead ECG findings that the absence of *P* waves was replaced by unorganized electrical activity and irregular R-R intervals^[7]^.

### Statistical Analysis

Analyses were conducted with the IBM Corp. Released 2011, IBM SPSS Statistics for Windows, version 20.0, Armonk, NY: IBM Corp. statistical analysis program. Data were presented as mean and standard deviation or median and interquartile range (IQR), number, and percentage. Normal distribution of continuous variables was evaluated with the Shapiro-Wilk W test when the sample size was < 50 and with the Kolmogorov-Smirnov test when the sample size was > 50. In comparisons between two independent groups, the independent samples *t*-test was used when normal distribution condition was met, and the Mann-Whitney U test was used if it was not. In comparisons between two dependent groups, a paired samples *t*-test was used when the normal distribution condition was met, and a Wilcoxon test was used if it was not. In 2 × 2 comparisons between categorical variables, the expected value (> 5) was calculated using Pearson’s Chi-square test; if the expected value was between 3 and 5, the Chi-square Yates test was used. The expected value (< 3) was attained using Fisher’s exact test. In the multivariate analysis, estimated risk factors between groups were assessed using logistic regression analysis employing the possible risk factors identified in previous analyses. Statistical significance was determined to be *P*<0.05.

## RESULTS

The 103 patients who met the specified criteria and underwent isolated CABG by the same surgical team were analyzed prospectively. Median age was 62 (IQR: 54-71) years, and 82 (79.6%) were male. The patients were divided into two groups: those who developed POAF (AF group [AFG]) and those who did not (non-AF group [NAFG]). POAF developed in 25 (24.3%) patients. The median age was 63 (55-74) years in AFG and 60 (54-68) in NAFG, and there was no statistically significant difference between the groups (*P*=0.137). However, the incidence of AF increased, especially in patients > 70 years. No statistically significant difference was observed between patient groups in evaluation of the demographic data summarized in Table 1.

**Table 1:** Relationship between demographic characteristics and POAF.

Demographic characteristics	NAFG (n: 78)	AFG (n: 25)	*P*-value
Age, years (median-IQR)	60 (54-68)	63 (55-74)	0.137
BMI (kg/m^2^) (mean±SD)	28.15±4.22	27.6±2.82	0.551
Sex, male, n (%)	63 (80.7)	19 (76)	0.607
DM, n (%)	33 (42.3)	8 (32)	0.360
Hypertension, n (%)	29 (37.1)	7 (28)	0.402
Cigarettes, n (%)	52 (66.6)	15 (60)	0.631

Independent samples *t*-test and Mann-Whitney U test

AFG=atrial fibrillation group; BMI=body mass index; DM=diabetes mellitus; IQR=interquartile range; NAFG=non-atrial fibrillation group; POAF=postoperative atrial fibrillation; SD=standard deviation

Preoperative data analyzed as potential predictors of POAF development are shown in Table 2. No statistically significant difference was observed between AFG and NAFG in the comparison of preoperative data. Preoperative PO₂ was measured as an average of 76 (58-98) mmHg in AFG patients and 80 (51-132) mmHg in NAFG patients. Although there was no statistically significant difference (*P*=0.164), it was observed that the frequency of POAF was clinically increased in patients with low preoperative PO₂ values.

**Table 2:** Preoperative variables and their effect on POAF.

Preoperative variables	NAFG (n: 78)	AFG (n: 25)	*P*-value
EF (%) (median-IQR)	55 (50-55)	55 (53-55)	0.206
Left atrial diameter (mm) (mean±SD)	34.8±3.14	35.88±3.66	0.194
Preoperative PO₂ (mmHg) (mean±SD)	80.02±11.99	75.92±10.31	0.164
Preoperative Hgb (g/dl) (mean±SD)	14.31±1.67	14.8±1.67	0.255
Preoperative Hct (%) (mean±SD)	42.8±4.71	43.16±4.55	0.860

Independent samples *t*-test and Mann-Whitney U test AFG=atrial fibrillation group; EF=ejection fraction; Hct=hematocrit; Hgb=hemoglobin; IQR=interquartile range; NAFG=non-atrial fibrillation group; PO2=partial pressure of oxygen; POAF=postoperative atrial fibrillation; SD=standard deviation

Intraoperative data on the development of POAF are presented in Table 3. All patients underwent isolated CABG under CPB. The median duration of CPB was 72 (IQR: 63-97) minutes in AFG and 82 (61-98) minutes in NAFG, and the difference was not statistically significant (*P*=0.717). The median CC time was recorded as 40 (32.5-48) minutes in AFG and 39.5 (30-46) minutes in NAFG. No statistically significant correlation was observed between CC time and AF (*P*=0.625). Left anterior descending artery distal anastomosis with LIMA was the only arterial graft used in 97% of patients. For other distal anastomoses, the vena saphenous magna was used as a graft. Five percent of the patients had two distal coronary anastomoses, 86% had three or four, and 9% had five. Graft numbers did not vary between the groups (*P*=0.177). The relationship between postoperative data and development of POAF is represented in Table 4, and no statistically significant difference was observed between groups.

**Table 3:** Intraoperative variables and development of POAF.

Intraoperative variables	NAFG (n: 78)	AFG (n: 25)	*P*-value
CPB duration (min) (median-IQR)	82 (61-98)	72 (63-97)	0.717
Cross-clamping time (min) (median-IQR)	39.5 (30-46)	40 (32.5-48)	0.625
Hypothermia (°C) (mean±SD)	32.05±0.27	32.2±0.57	0.125
Number of grafts (median-IQR)	4 (3-4)	3 (2.5-4)	0.177
LIMA, n (%)	76 (97.4)	24 (96)	0.570
RCA, n (%)	54 (69.2)	19 (76)	0.517

Independent samples *t*-test and Mann-Whitney U test AFG=atrial fibrillation group; CPB=cardiopulmonary bypass; IQR=interquartile range; LIMA=left internal mammary artery; NAFG=non-atrial fibrillation group; POAF=postoperative atrial fibrillation; RCA=right coronary artery; SD=standard deviation

**Table 4:** Postoperative factors and their relationship with POAF.

Postoperative variables	NAFG (n: 78)	AFG (n: 25)	*P*-value
Postoperative EF (%) (mean±SD)	51.38±6.58	53.2±5.18	0.134
Postoperative PO₂ (mmHg) (mean±SD)	119.53±20.66	118.72±21.71	0.988
Postoperative Hgb (g/dL) (mean±SD)	8.71±0.76	8.72±0.8	0.784
Postoperative Hct (%) (mean±SD)	26.3±2.45	25.86±2.43	0.292
Intubation time (hours) (mean±SD)	4.43±2.49	4.64±2.17	0.483
Intensive care unit stay (days) (mean±SD)	2.66±0.71	2.88±1.01	0.534
Na (mmol/l) (mean±SD)	142.2±4.13	142.6±3.73	0.832
K (mmol/l) (mean±SD)	4.11±0.38	4.12±0.35	0.945
Ca (mg/dl) (mean±SD)	8.48±0.63	8.7±1.08	0.972
Creatinine (mg/dl) (mean±SD)	0.93±0.2	1±0.23	0.157

Independent samples *t*-test and Mann-Whitney U test AFG=atrial fibrillation group; Ca=calcium; EF=ejection fraction; Hct=hematocrit; Hgb=hemoglobin; K=potassium; Na=sodium; NAFG=non-atrial fibrillation group; PO2=partial pressure of oxygen; POAF=postoperative atrial fibrillation; SD=standard deviation

In addition, there was a negative correlation between CC time and preoperative EF (r = -0.264, *P*=0.007), postoperative Hct (r = -0.246, *P*=0.012), and postoperative hemoglobin (r = -0.209, *P*=0.034), as well as between CPB time and preoperative EF (r = -0.287, *P*=0.003) and postoperative Hct (r = -0.217, *P*=0.028).

## DISCUSSION

In this study, we aimed to examine the effects of CC duration on development of POAF. Our secondary aim was to examine the effects of preoperative, operative, and postoperative variables on development of AF. Similar to the literature, we observed 24.3% POAF, and it mostly occurred within the first three days. We did not observe a statistically significant relationship between potential risk factors and development of POAF. However, the increased rate of POAF in the advanced age group was significant. Consequently, we did not observe a statistically significant relationship between duration of CC and development of POAF.

Incidence of POAF after open-heart surgery has a wide range depending on the type of operation and the morbidity factors of the patient^[1]^. Widely observed risk factors affecting development of POAF include advanced age, preoperative cardiac arrhythmias, congestive heart failure, ischemic heart disease, preoperative use of intra-aortic balloon pump (IABP), and obesity^[8]^. LA enlargement is a common finding in severe obesity that predominantly affects cardiopulmonary physiology^[9]^. Perrier et al.^[10]^ determined that severe obesity (BMI ≥ 35) was an independent preoperative predictor of POAF. In our study, no significant difference was observed between AFG and NAFG cases regarding BMI (27.6 ± 2.82 kg/m2 and 28.15 ± 4.22 kg/m^2^, respectively, and *P*=0.551). Similarly, a recent study by Mangi et al.^[11]^ measured BMI as a mean of 26.88 ± 5.16 kg/m^2^, and no statistically significant association was observed between high BMI and POAF after isolated CABG.

Advanced age is the most important and consistently proven risk factor for development of POAF. Indeed, the risk of POAF increases 1.5-fold for every five-year increase from age 50 to 75 years^[12]^. Similarly, a large, multi-center, prospective observational study involving 4,600 patients showed that each 10-year increase in age raises the probability of developing AF by 75%. Hence, based on age alone, anyone > 70 years is at increased risk for developing AF^[4]^. This increased risk of AF developing with age may be associated with age-related changes in the atrial connective tissue, dilatation, and non-uniform anisotropic conduction^[4,13]^. We found that age was not statistically significant between AFG and NAFG cases (*P*=0.137). However, we believe that the high median age in AFG - that is, 63 (IQR: 55-74) *vs.* 60 (54-68) years - and the increased frequency of POAF in patients > 70 years are clinically significant.

While the number of distal anastomoses may be one of the parameters indicating the severity of coronary artery disease, it might be a risk factor because it might increase myocardial dysfunction and prolong the duration of CC. In our study, the mean number of distal anastomosis was found to be three (IQR: 2.5-4) in AFG and four (3-4) in NAFG, and no statistically significant difference was observed between groups (*P*=0.177). Similarly, Rubin et al.^[14]^ revealed that the number of coronary artery lesions was not effective in the formation of POAF.

Wolfe et al.^[15]^ illustrated that POAF increases mortality and morbidity after CABG. Creswell et al.^[16]^ demonstrated the effects of POAF on postoperative morbidity and mortality. Maisel et al.^[17]^ revealed that POAF after CABG prolongs ICU and hospital stay, thus raising treatment costs. Another study found that POAF can cause hemodynamic instability and symptoms of congestive heart failure, lengthening stays in the ICU and hospital, and, therefore, increasing costs^[3]^. We observed no disparity between AFG and NAFG patients regarding their length of stay in the ICU or hospital. Prolonged postoperative ventilation is also known to be an independent risk factor for POAF^[11]^. We also found that the duration of intubation was similar in both groups (*P*=0.483) and was not associated with POAF.

A recent review by Jannati mentioned that the etiology of POAF does not depend on a specific factor and that several factors may lead to the incidence of POAF^[18]^. Potential factors played a role in creating atrial susceptibility to POAF: pericarditis, atrial injury from surgical trauma or cannulation, atrial suture lines, acute atrial enlargement due to pressure or volume overload, insufficient myocardial protection during CPB, atrial ischemia, long CPB and aortic CC durations, hyperadrenergic conditions (such as postoperative use of inotropic drugs), pulmonary complications, hypoxemia, inflammation, hypokalemia, and hypomagnesemia^[18]^. Sabzi et al.^[19]^ analyzed the data of 700 patients retrospectively, investigated approximately 31 parameters, classified these parameters as preoperative, intraoperative, and postoperative, and tried to determine the risk factors’ relationship with POAF. Among the intraoperative variables, duration of CC, duration of CPB, use of LIMA, type of operation, use of inotropic support, use of IABP, and number of grafts were examined, and the long duration of CC and use of IABP were associated with POAF^[19]^. In 2012, Ben Ahmed et al.^[20]^ found that long CC duration was significant to development of POAF (77.6 min *vs.* 59.8 min; *P*=0.009). Contrary to these studies, we did not observe a statistically significant difference between AFG and NAFG patients concerning the duration of CC and CPB (*P*=0.625; *P*=0.717; respectively). Similarly, İsmail et al.^[3]^ found no significant difference in duration of CC, total duration of CPB, or degree of hypothermia between patients with and without POAF. The findings of Rajabi et al.^[5]^ were similar to those of our study. Their variables, such as EF, sodium, potassium, duration of CC, duration of CPB, and intubation, did not affect the incidence of POAF (*P*>0.05). The most striking point in these studies was the length of aortic CC and CPB times. When these periods are within reasonable limits, they do not affect the development of POAF; however, the incidence of POAF increases especially when duration of CC is > 60 minutes and duration of CPB is > 100 minutes^[21]^.

The effect of extracorporeal circulation on the development of AF after isolated CABG remains a controversial issue. In the largest randomized controlled trial to date, to the best of our knowledge, comparing outcomes between on-pump and off-pump CABG procedures in 4,752 patients, no difference in the development of POAF was observed between the two groups^[22]^. In a prospective cohort study in which Rostagno et al.^[6]^ investigated the long-term effects of the incidence of AF after isolated CABG and its relationship with operative technique in 2014, 229 patients were included. The frequency of POAF did not vary between patients who underwent on-pump and off-pump CABG. Similar to our study, these studies support the hypothesis that CBP and CC times do not affect POAF, since there is no significant difference between the two surgical techniques in development of POAF^[6,22]^. However, Hashemzadeh et al.^[23]^ showed that the development of POAF was significantly reduced after off-pump CABG compared to on-pump CABG, and the conversion from POAF to a sinus rhythm was likelier in patients who underwent off-pump CABG. Similarly, some meta-analyses revealed lower rates of POAF in patients who underwent off-pump CABG^[24,25]^.

### Limitations

This study has some limitations, the major limitation was an insufficient sample size and, hence, the inability to exclude type 2 errors. Second, all patients were provided β-blockers to take before surgery; accordingly, the incidence of POAF may be low due to its protective effect. Third, the cases were operated on by the same team and had a homogeneous structure with relatively low CPB and CC time averages. Therefore, the effect of prolonged CPB and CC times may not have been correctly evaluated.

## CONCLUSION

POAF is one of the most common and most important arrhythmias that develop after isolated CABG. Many factors affect the development of POAF. Although many studies have been conducted to understand its etiology and prevent its development, there is no definite consensus on the factors affecting POAF. Despite significant advances in surgery and perioperative care in the last two decades, no reduction in incidence of POAF or associated complications has been achieved in clinical practice. In this respect, the subject maintains its currency and importance. Considering the information we obtained because of our study, it has been shown that there is no significant relationship between duration of CPB and CC and incidence of POAF. However, there is still a need for large-scale, multicenter clinical studies on the subject.

**Table:** 

Authors’ Roles & Responsibilities	
HID	Substantial contributions to the conception or design of the work; or the acquisition, analysis, or interpretation of data for the work; drafting the work or revising it critically for important intellectual content; agreement to be accountable for all aspects of the work in ensuring that questions related to the accuracy or integrity of any part of the work are appropriately investigated and resolved; final approval of the version to be published
ESÇ	Substantial contributions to the conception or design of the work; or the acquisition, analysis, or interpretation of data for the work; drafting the work or revising it critically for important intellectual content; agreement to be accountable for all aspects of the work in ensuring that questions related to the accuracy or integrity of any part of the work are appropriately investigated and resolved; final approval of the version to be published
OB	Agreement to be accountable for all aspects of the work in ensuring that questions related to the accuracy or integrity of any part of the work are appropriately investigated and resolved; final approval of the version to be published
MEA	Substantial contributions to the conception or design of the work; or the acquisition, analysis, or interpretation of data for the work; drafting the work or revising it critically for important intellectual content; agreement to be accountable for all aspects of the work in ensuring that questions related to the accuracy or integrity of any part of the work are appropriately investigated and resolved; final approval of the version to be published
FB	Agreement to be accountable for all aspects of the work in ensuring that questions related to the accuracy or integrity of any part of the work are appropriately investigated and resolved; final approval of the version to be published
ZY	Agreement to be accountable for all aspects of the work in ensuring that questions related to the accuracy or integrity of any part of the work are appropriately investigated and resolved; final approval of the version to be published
BE	Agreement to be accountable for all aspects of the work in ensuring that questions related to the accuracy or integrity of any part of the work are appropriately investigated and resolved; final approval of the version to be published
YÜ	Agreement to be accountable for all aspects of the work in ensuring that questions related to the accuracy or integrity of any part of the work are appropriately investigated and resolved; final approval of the version to be published
